# The effect of carotenoid supplementation on immune system development in juvenile male veiled chameleons (*Chamaeleo calyptratus*)

**DOI:** 10.1186/1742-9994-11-26

**Published:** 2014-03-22

**Authors:** Kristen L McCartney, Russell A Ligon, Michael W Butler, Dale F DeNardo, Kevin J McGraw

**Affiliations:** 1 School of Life Sciences, Arizona State University, Tempe, AZ 85287-4501, USA; 2 Current Address: 118 Milton Ave, Chapel Hill, NC 27514, USA; 3 Current Address: Department of Biology, Lafayette College, 213 Kunkel Hall, Easton, PA 18042-1778, USA

**Keywords:** Antioxidant, Innate immunity, Nitric oxide, Reptiles, Wound healing

## Abstract

**Introduction:**

Nutrient availability, assimilation, and allocation can have important and lasting effects on the immune system development of growing animals. Though carotenoid pigments have immunostimulatory properties in many animals, relatively little is known regarding how they influence the immune system during development. Moreover, studies linking carotenoids to health at any life stage have largely been restricted to birds and mammals. We investigated the effects of carotenoid supplementation on multiple aspects of immunity in juvenile veiled chameleons (*Chamaeleo calyptratus*). We supplemented half of the chameleons with lutein (a xanthophyll carotenoid) for 14 weeks during development and serially measured multiple aspects of immune function, including: agglutination and lysis performance of plasma, wound healing, and plasma nitric oxide concentrations before and after wounding.

**Results:**

Though lutein supplementation effectively elevated circulating carotenoid concentrations throughout the developmental period, we found no evidence that carotenoid repletion enhanced immune function at any point. However, agglutination and lysis scores increased, while baseline nitric oxide levels decreased, as chameleons aged.

**Conclusions:**

Taken together, our results indicate that body mass and age, but not carotenoid access, may play an important role in immune performance of growing chameleons. Hence, studying well-understood physiological processes in novel taxa can provide new perspectives on alternative physiological processes and nutrient function.

## Introduction

Resource availability, assimilation, and allocation can affect the development and function of multiple physiological processes, including those of the immune system [1]. While the capability to eliminate pathogens and recover from an immune challenge is critical to survival throughout life, robust immune function is particularly important to juveniles. Juveniles must survive the energetically demanding developmental phase to reach sexual maturity, and do so in sufficient health to reproduce [2]. Additionally, exposure to pathogens during development can enhance immune function in adulthood (e.g., through acquired immunity; [3]). Because of the costs associated with immune responses, however, the immune system is likely to consume resources at the expense of other costly physiological processes, such as growth and development [2,4], ornamentation [5], and reproduction [6]. Though numerous studies (e.g. [4,5,7,8]; reviewed in [9]) have documented trade-offs between growth and immune system development in juveniles, the limiting resources (e.g. energy, nutrients) and their influence on development are likely to vary among taxa.

One well-studied physiological trade-off occurs with carotenoids, which, for vertebrates, can only be acquired through the diet [10]. Carotenoids are a class of organic compounds that have bright red, orange, and yellow hues [11] and can produce colorful ornamental structures [12-16], but are simultaneously used for vital physiological processes [17-19]. Significantly, carotenoid molecules can play an important role in both the activation and regulation of the immune system (reviewed in [20]), though these relationships are not universal [21]. For example, carotenoids can enhance antibody production [22] and activation of the plasma complement cascade (a set of proteolytic enzymes [23]) that promote inflammation, as well as the agglutination and marking of invasive microbes for destruction by lysis or phagocytosis [24]. Additionally, immune responses are frequently linked to increased oxidative stress [25], illustrating the potential for a mechanistic link between antioxidants (such as carotenoids) and immunity. For example, the antioxidant activity of carotenoids has been implicated in ameliorating damage caused by free radicals, such as nitric oxide (NO), produced by immune cells when responding to pathogenic threats (reviewed in [20,26]). Carotenoids can also stimulate attacks on foreign cells after the skin has been breached (by activating an unidentified feature of the acquired immune system [27]), and may facilitate rapid recovery of wounds, as has been shown for other antioxidants [28]. Recovery from physical wounds is vital, because animals that heal wounds more quickly can minimize time spent in a vulnerable state [29,30], yet this aspect of immune response is rarely considered. After wounding, carotenoids (in their role as antioxidants [20,31]) may even shield important host cells and structures from circulating NO [32] which is released as a defense against pathogens in response to inflammation [33-35], yet is indiscriminately destructive to foreign microbes and host cells alike [36,37].

**Figure 1:**
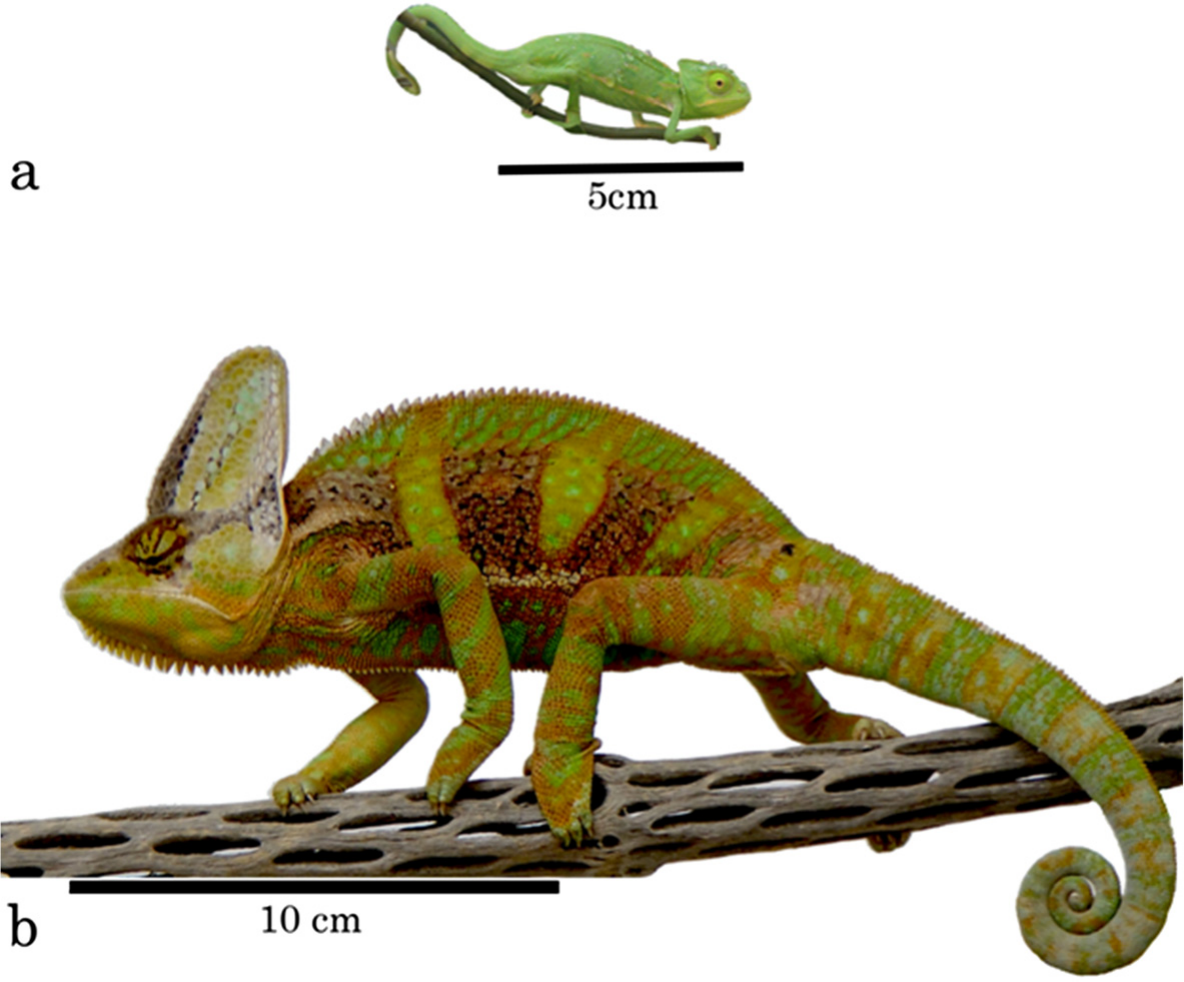
**Juvenile veiled chameleons (*Chamaeleo calyptratus*) during (a) Week 1 (approximately 8 weeks of age) and (b) Week 17 of the study.**

The majority of literature on carotenoid physiology in animals has focused on the antioxidant functions and immunoregulatory roles of these compounds in birds [21], despite their demonstrable physiological role in other taxa (e.g., mammals [38], reptiles [39], fish [23]). Given the relative lack of taxonomic diversity in carotenoid physiology research and the idea that dietary access to these nutrients during development (including embryonic stages [40]) may be particularly important for developing immune systems (e.g. [41]), we tested the hypothesis that carotenoid supplementation during a period of rapid growth and development would improve immune system performance of captive juvenile male veiled chameleons (*Chamaeleo calyptratus*; Figure 1). Veiled chameleons are a reptile species that uses carotenoids in their dynamic and colorful displays as adults (authors’ unpublished data), though the pigmentary content of the predominantly green coloration of juveniles is unknown. Additionally, veiled chameleons demonstrate intense intrasexual aggression and combat as adults [42] and as juveniles (pers. obs.). This agonistic behavior occasionally results in open wounds (authors’ unpublished data), which could provide additional selective pressure for the ability to quickly heal injuries (which can also be obtained in a number of other ways, for instance via predation attempts). Therefore, we used a standardized biopsy procedure to quantify wound healing, a biologically relevant measure of multiple phases of the immune response [30], during development. The biopsy simultaneously provided an immune-challenge to our chameleons that could be used to monitor other metrics of immune function in blood samples. Blood was measured for circulating plasma NO and carotenoid levels, as well as the agglutination ability of antibodies and the lytic capacity of complement (quantified by a sheep red blood cell (SRBC) assay). We predicted that dietary carotenoid supplementation would result in elevated levels of circulating carotenoids, which in turn would positively affect various aspects of immunity and wound healing. Specifically, both within and between treatment groups, we predicted that higher carotenoid levels would be associated with a greater ability of circulating antibodies and complement to agglutinate and lyse SRBCs [23]. We also predicted that higher levels of circulating carotenoids would have a two-pronged effect on NO levels and wounding healing: (i) in their role as immunostimulants (reviewed in [20]), carotenoids should initiate and accelerate the NO burst following wounding, and (ii) by serving as antioxidants [20,43,44], carotenoids should counteract the indiscriminately destructive pro-oxidant activity of NO, allowing carotenoid-supplemented individuals to avoid self-damage associated with NO and lead to faster rates of wound healing [44].

## Methods

### Study design

We purchased two groups of 10, eight-week old male veiled chameleon siblings through a private breeder (FL Chams, Florida, USA) in July 2011. Chameleons were initially housed in groups of five brothers in four identical cages (89 × 56 × 53 cm) with screen roofs and doors, feeding cups, and automated misters (which misted for three minutes, four times per day). All chameleons experienced a 12:12 light:dark cycle, and we provided each cage with a heat lamp (Zoo Med Repti-Basking Spot Lamp, 50 watt, Zoo Med laboratories Inc., San Luis Obispo, CA, USA) and a UV light source (Zoo Med Reptisun 5.0 UVB Fluorescent Bulbs, Zoo Med laboratories Inc., San Luis Obispo, CA, USA). Individual cage lamps were set to turn on 15 min after the room lights to simulate dawn and turn off 15 min before room lights to simulate dusk. We housed the chameleons in an indoor, climate-controlled room with the daily temperature range set to 25.0-27.2°C and an average humidity of 25-35%.

All cages were furnished with grape vines (*Vitis* spp.), yellow palo verde (*Parkinsonia microphylla*) branches, and faux vines to provide perches at various heights for thermoregulation under the heat lamps [45]. Space limitations necessitated that we place chameleon cages in two tiers, with one group of 10 brothers (hereafter, family A) occupying the upper cages of our stacked racks and the remaining group of brothers (family B) occupying the lower cages. All members of each family were kept at the same relative height throughout the duration of the study to minimize within-family differences in housing conditions and maximize our ability to detect any effects of carotenoid supplementation. After an eight week acclimation period, we moved all chameleons into individual cages to prevent excessive aggression [45,46]. During both the acclimation and experimental periods, we fed the chameleons crickets dusted with calcium (*Rep-Cal* Phosphorus-free Calcium, 0% D3, Rep-Cal Research Labs, Los Gatos, CA, USA) and vitamins (Zoo Med Reptivite Reptile Vitamins, Zoo Med Laboratories Inc., San Luis Obispo, CA, USA) throughout the study. Wild chameleons have access to a broader array of dietary items, so the limited diversity of commercially available food items necessitates dietary supplements such as these [46]. Reptivite vitamins contained no measurable quantities of any carotenoid.

### Experimental design

Four weeks into the eight-week acclimation period, we acquired pre-supplementation baseline values for wound healing, agglutination and lysis, and plasma carotenoid levels (see below) and randomly assigned half of each group of brothers to the carotenoid-supplemented (C) group and the remaining animals to the control group (non-supplemented = N). This arrangement resulted in four distinct groups, two supplemented family groups (AC and BC) and two control family groups (AN and BN). During weeks 8-21 of the study, supplemental carotenoids were given twice weekly in the form of an oral administration of powdered 10% lutein (FloraGLO Lutein 10% CWS/S-TG, Kemin Industries, Inc., Des Moines, IA) dissolved in 1 mL of warm (~ 46°C) distilled water. Warm water was required to break down the starch-based transport molecules containing the carotenoids and create a homogenous solution for oral supplementation. To control for potential effects of handling stress and hydration state, the control group received oral supplementations of 1 mL of warm distilled water. We weighed each chameleon to the nearest 0.01 g with an electronic balance each time we administered the carotenoid and water solutions.

### Determination of carotenoid supplementation levels

Given the dearth of literature regarding the carotenoid content in circulation or in the diet of wild veiled chameleons, we identified and quantified the carotenoids present in a selection of arthropods collected from LaBelle, FL, USA, in a habitat known to contain a feral population of veiled chameleons, as a means of determining an appropriate dietary carotenoid dose to administer in our experiment (Additional file 1: Table S1). We desiccated the dead arthropods in a 40°C drying oven overnight before pulverizing them (first with a mortar and pestle, then a micronizer) in ethanol until homogenized; from this homogenate, we then extracted carotenoids as described below [47]. We found that lutein made up the largest portion (28%) of total carotenoids in the wild arthropods and on average made up 48.20 (± s.e. 21.10) μg/g of a food item. Additionally, lutein was the predominant carotenoid circulating in the plasma of unmanipulated chameleons, representing 88.6% of the total circulating carotenoids. Therefore, to elevate carotenoid levels above those in a typical chameleon diet, we administered a concentration of lutein that corresponded to one standard deviation (63.30 μg/g) above this average (48.20 + 63.30 = 111.50 μg/g), calculated using the average mass of crickets eaten per day per individual in our captive colony. Initially, we provided all chameleons 10 half-inch crickets per day (average cricket mass = 76.68 mg, average total mass consumed = 766.79 mg), but as the chameleons grew and regularly consumed all food items provided we increased this to 15 adult crickets per day (average mass = 288.61 mg, average total mass consumed = 4.33 g) in Week 10. Therefore, for the first two weeks of treatment (Weeks 8-10), the chameleons received a dose of 854.93 μg of 10% lutein in 1 mL of water (an effective dose of 85.49 μg of lutein), and thereafter were given 4826.70 μg of 10% lutein in 1 mL (an effective dose of 482.67 μg of lutein). For reference, previous studies examining the effects of carotenoid supplementation on lizards have typically administered lower levels of carotenoids (e.g. 0.084 ug/supplementation for brown anoles *Norops sagrei*[48] and 20 ug/supplementation for Australian painted dragon lizards *Ctenophorous pictus*[49]).

### Blood collection

**Figure 2:**
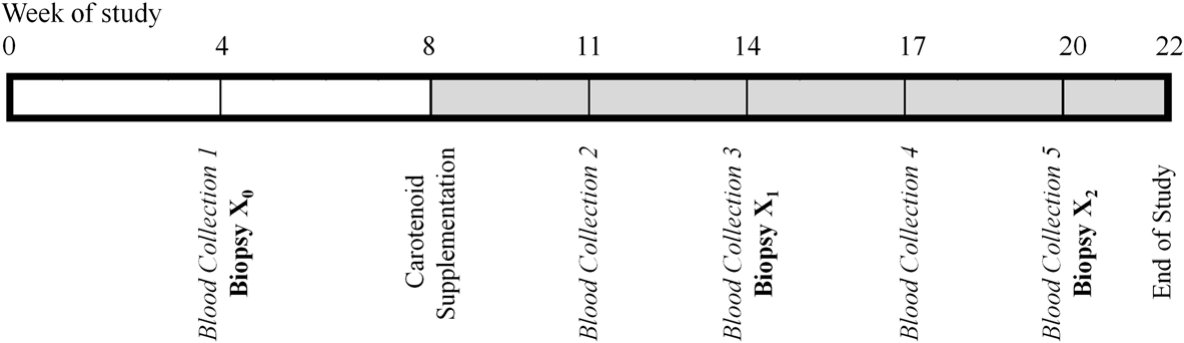
**Timeline of carotenoid supplementation, blood collection, and dermal biopsies during our study.** During Week 4 of the study, blood was collected from all individuals to establish initial values for circulating carotenoid, agglutination, and lysis titers prior to supplementation (*Blood Collection 1*). Additionally, biopsy X_0_ was performed during Week 4 of the study to determine the post-wounding time points with the greatest variation in wound area (to focus subsequent wound healing analyses). In Week 8 of the study, carotenoid supplementation commenced. Every three weeks during the supplementation period, blood was collected to measure circulating carotenoid levels and agglutination and lysis titers (*Blood Collections 2-5*). At weeks 14 and 20 of the study, we biopsied (X_1_, X_2_) the chameleons to evaluate wound healing. Wound healing rates were determined by measuring wound area from photographs taken immediately after biopsies, and on days 6 and 10 after wounding. Furthermore, we evaluated circulating nitric oxide levels by collecting blood samples immediately before, and again two days, after each biopsy.

To assess circulating carotenoid levels and some aspects of immunity, we drew blood five times during the study – once prior to carotenoid supplementation (Week 4) and at four other times during the supplementation period (Weeks 11, 14, 17, and 20; Figure 2). At each collection point, we collected up to 0.1% of each chameleon’s body mass of blood from the caudal vein with a 0.3 cc heparinized syringe and kept this blood on ice until centrifugation (5 min at 10,000 rpm). After centrifugation, we drew off plasma and stored it at -80°C for subsequent analyses (see below).

### Carotenoid extraction and analysis

High-performance liquid chromatographic analyses of carotenoids followed previously published methods [47], with the following modifications. We sequentially extracted carotenoids from 20 μL of thawed plasma with 200 μL of both ethanol and 1:1 hexane:tert-butyl methyl ether, then vortexed the extract briefly before centrifugation for 3 min at 10,000 rpm. We transferred the supernatant to a fresh, labeled tube and then evaporated it to dryness under nitrogen and stored it at -80°C. We resuspended samples in 200 μL of HPLC mobile phase (42:42:16, v/v/v, methanol:acetonitrile:dicholoromethane) and vortexed them until all pigment was visibly suspended. By comparing their retention times and absorbance maxima to external standards and published specifications [14], we identified the following carotenoid pigments in chameleon plasma: lutein, cis lutein isomers, zeaxanthin and three canary xanthophylls (A-C). We determined pigment concentrations by integrating the peaks and by comparing these areas to a standard curve. Concentrations of all carotenoid types were significantly and positively correlated with each other (all r > 0.76, *P* < 0.0001, Additional file 2: Table S2), indicating that our supplementation of lutein also influenced circulating levels of all measured carotenoid types. Therefore, we used total circulating carotenoid concentration in all subsequent analyses to better understand the general importance of carotenoids, as opposed to just lutein, on immunity in growing chameleons.

### Agglutination and lysis

To examine the effect of carotenoid supplementation on immune system performance in juvenile chameleons, we used a modified version of the agglutination and lysis assay described by Matson et al. [50], which is a highly repeatable and informative way to measure the strength of the humoral components of the constitutive innate immune system (specifically, antibodies and complement enzymes; [50]). We made two modifications to the procedure described by Matson et al. [50] based on pilot work to maximize our ability to detect inter-individual variation: 1) we reduced the concentration of the SRBC suspensions in this assay from 1% to 0.5% and 2) the incubation temperature was decreased from 37°C to 26.5°C. This temperature change allowed the assay incubation to more accurately reflect the ambient temperature in which study animals were housed (range 25.0 - 27.2°C), as has been done previously with ectotherms [51] and which is important because incubation temperature plays a significant role in agglutination and lysis [52]. After incubation for 90 min, the plates were tilted for 20 min at 45° to permit visualization of the agglutination response, then digitally scanned at a resolution of 600 dpi. After scanning, the plates were laid out on a flat surface for 70 min before scanning a second time to record the lysis response. This assay thus identifies the plasma dilution at which agglutination (clumping of sRBC) and lysis (destruction of sRBC) occur due to innate immune mechanisms. The assessments of two independent investigators, blind to treatment, family, and individual, were significantly repeatable [53] for both agglutination (*r* = 0.990*, P* < 0.0001) and lysis (*r* = 0.989*, P* < 0.0001), so we used the average of these scores for all subsequent statistical analyses. Because agglutination and lysis were highly positively correlated and are at least partially mechanistically linked [50], we performed principal components analyses (PCA) to collapse agglutination and lysis scores into a single metric (with two metrics, this is the covariation between the two). The agglutination and lysis scores for chameleons during development reduced to a single principal component that explained 98% of the variation among these variables (agglutination loading, 0.71, lysis loading, 0.71). Therefore, individuals with high positive PC1 scores exhibited strong agglutination and lysis responses.

### Wound healing

We took dermal biopsies from each chameleon at three time points during the study and recorded changes in wound area using digital photography [30] and image processing and analysis freeware (ImageJ64, NIH [54]). We collected dermal biopsies using a standardized procedure based on a previous wound healing study [30], though we modified biopsy placement to better suit chameleon morphology. Immediately after blood collection, we anesthetized each chameleon using isoflurane, then disinfected the biopsy site with isopropyl alcohol and removed a circular section of the epidermis and dermis near the hip using a 3.5 mm biopsy punch. After wounding, pressure was applied for three minutes or until bleeding was stopped before the wound was photographed. We conducted the first biopsy (X_0_) and all associated wound photography (Weeks 4-7) before carotenoid supplementation began. We performed two other biopsies adjacent to, but not at, the location of previous biopsies during the supplementation period at predetermined time points corresponding to the middle (Biopsy 1 = X_1_; Week 14) and end (Biopsy 2 = X_2_; Week 20) of the study. The second biopsy (X_1_) was conducted on the opposite side of the body as the first biopsy (X_0_), and the third biopsy (X_2_) was on the same side as biopsy X_0_, separated by approximately 8 mm.

All wounds were photographed in a standardized fashion to control for angle and distance, as well as to improve repeatability. Chameleons were manually restrained during photography sessions and all photographs were taken with a digital camera (Panasonic HDC-TM700P/PC, Panasonic Corporation, Kadoma, Osaka, Japan) on a small tripod. We positioned light sources to best illuminate the wound area and elevated a ruler to be level with the wound for setting scale in later image analysis. A minimum of four photographs were taken of each wound, ensuring at least two were zoomed in as close as possible without compromising focus.

After X_0_, we monitored and photographed all wounds every other day, beginning on the day of biopsy (D_0_), until at least one chameleon’s wound was fully healed (i.e., biopsy site was completely covered in smooth, gray scar tissue or new colorful epidermis). From a subset of these photographs, we determined two time-points (Day 6 = D_6_ and Day 10 = D_10_) during the healing process that yielded the greatest variation in wound size, suggesting that wound measurements taken on these days during future biopsies would be most likely to capture inter-individual variation in wound healing rate.

All wound photographs were viewed in ImageJ64 to assess the size of the wound (mm^2^), with the investigator blind to sampling date, treatment, and chameleon identity (ImageJ64, NIH [54]). Based on French et al.’s [30] study in tree lizards (*Urosaurus ornatus*), we used the area of the wound as a measure of wound healing (where area was the most reliable metric because wounds healed in a non-uniform manner and irregular closure of wounds was common). Specifically, the area of any portion of the biopsy site that was considered unhealed “wound” (i.e., textured or deep red) was measured (Additional file 3: Figure S1). On every occasion of wound measurement, at least two photos with both a well-focused ruler and wound were analyzed and the wound areas averaged. We calculated the repeatability [53] of wound sizes during one round of wound healing and found that wound size (mm^2^) was significantly repeatable for all chameleons (*r* = 0.97, *P* < 0.001).

### Nitric oxide assays

We analyzed the change in NO during the course of each wounding to measure the magnitude of the chameleon innate immune response [55]. We collected blood immediately before wounding (and anesthetization) and two days after wounding, in an attempt to sample animals near the peak of the NO burst (as determined by [34] in mammals), and stored 50 μL of the plasma fraction at -80°C until NO assays could be performed. The NO assay was conducted as described by Butler and McGraw [56], adapted from [55]. We first deproteinized 15 μL of plasma in 40 μL of zinc sulfate (75 mM) and 50 μL of sodium hydroxide (55 mM), then centrifuged the samples for 10 min at 16,000 RPM. We combined 80 μL of the supernatant with 80 μL of glycine buffer (pH 9.7) before adding two activated cadmium granules and vortexing the samples for 15 min. Next, we combined 120 μL of supernatant with 120 μL of Greiss reagent (Sigma G4410-10G) before vortexing for another 15 min. Finally, we transferred 200 μL of each sample into a 96-well plate and used an iMark Microplate Reader (Bio-Rad Laboratories, UK) to measure the absorbance of each well at λ = 540 nm. Using the standard curves run with each assay, we converted absorbance values to concentrations of plasma NO.

### Statistical analyses

To test if circulating carotenoid levels, body mass, NO levels, agglutination and lysis titers, and wound-healing rate differed as a function of carotenoid treatment, family, age, and all interactions, we ran repeated-measures analysis of variance (rmANOVA) models. Because we were interested in the relationship between circulating carotenoid levels and NO levels, agglutination performance, and lysis performance within individuals regardless of dietary access to carotenoids, we also ran *post hoc* analysis of covariance (ANCOVA) models within each treatment group and sampling period, with circulating carotenoid levels as the covariate, family as a fixed effect, and NO levels and agglutination and lysis PC1 values as dependent variables. Because of potential dilution effects of circulating carotenoids as body size increases [57], we tested whether body mass affected circulating carotenoid concentration by performing simple linear regressions within each treatment group. We were also interested in the relationship between body mass and circulating carotenoid levels, and agglutination and lysis titers; however, body mass of individuals was significantly different between families (see below). Therefore, to ensure that both mass and family terms were not explaining the same variance, we ran *post hoc* ANCOVAs within each sampling period and family, with mass as the covariate, treatment as the independent variable, and wound size and agglutination and lysis titers as dependent variables. We used SAS 9.2 (Cary, NC) for all analyses and, when the assumption of sphericity was violated (Greenhouse-Geisser Epsilon < 0.7), we interpreted the G-G adjusted p-value (*P*).

For most of the study, every chameleon was measured at every sampling period. However, an unanticipated infection forced us to eliminate one individual (group AC) from the Week 17 blood collection and biopsy 2. Additionally, problems with photo clarity and wound scabbing prohibited accurate wound area measurement in some photographs, resulting in the elimination of two individuals from biopsy X_0_ (one each from groups BC and BN) and four individuals from biopsy X_1_ (one from AC and three from BC); hence we had to eliminate each of these six animals from our full repeated-measures analysis. Thus, we ran a mixed-models analysis for comparison; unlike rm-ANOVA, mixed-models allow for the incorporation of all data points from a subject, even if observations are missing for that subject.

## Results

### Effects of family and treatment on body mass throughout development

**Figure 3:**
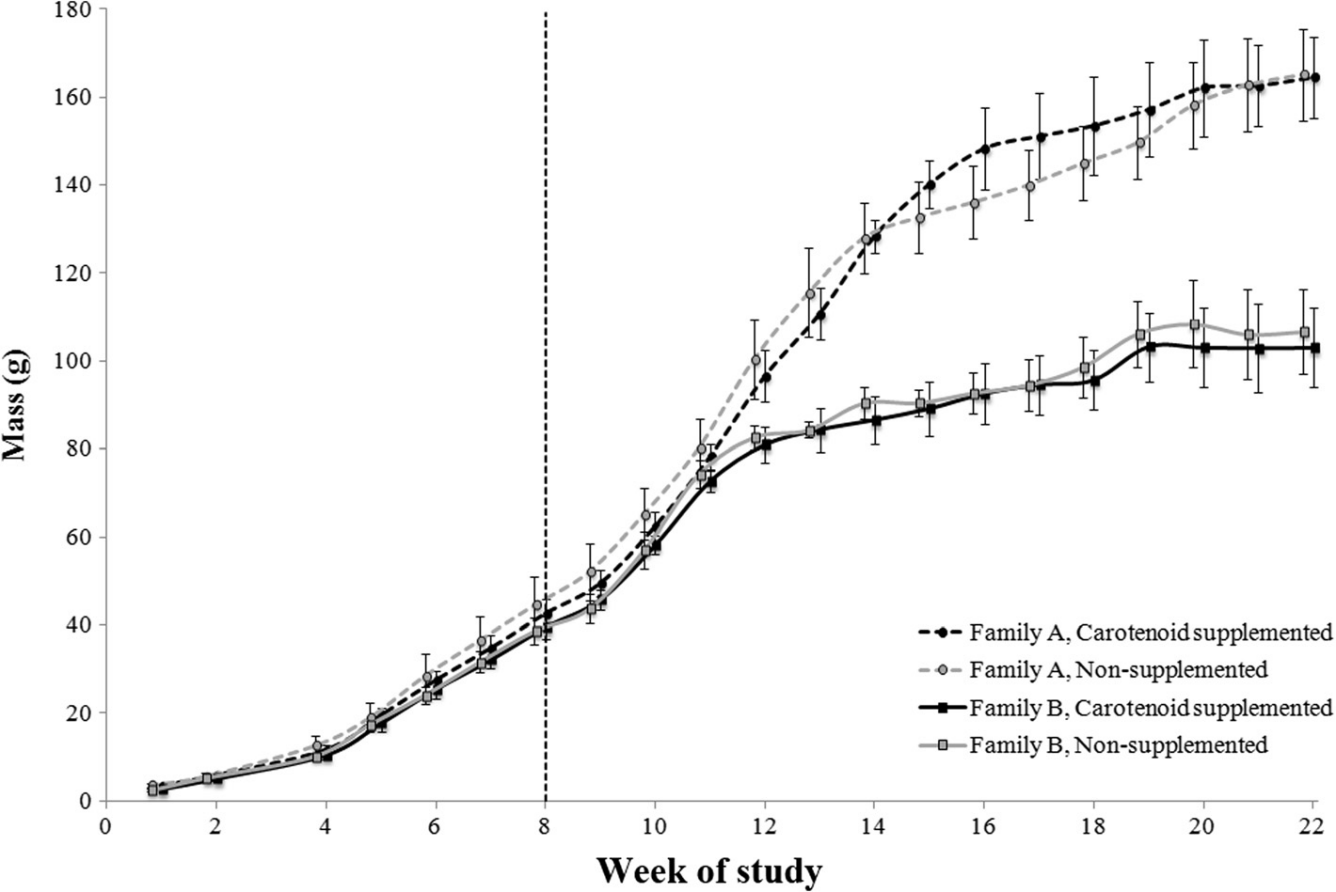
**Weekly body masses of growing veiled chameleons during development.** Measurements for all groups were taken at the same times, but data points are slightly offset for ease of interpretation. The vertical dashed line (at Week 8 of the study) represents the onset of carotenoid supplementation. All chameleons were provided with the same number of food items each day. Family A was significantly more massive than Family B at Week 1 and from Week 12 through Week 22 of the study. Points represent mean values with standard errors calculated from raw data.

Mass did not significantly differ by treatment at any age (all *F*_1,16_ < 0.64, all *P* > 0.4), though family groups differed in the degree to which chameleons gained body mass over time (Week*Family *F*_20,320_ = 28.02, *P* < 0.0001). Mass was significantly different between families at Week 1 (*F*_1,16_ = 11.76, *P* = 0.003). This difference between families did not exist from Week 2 through Week 11 (all *F*_1,16_ < 2.33, and all *P* > 0.146), but at Week 12 a significant difference in mass between families arose that persisted until the end of the study (all *F*_1,16_ > 7.87, all *P* < 0.0127; Figure 3). Overall there was a significant effect of family on mass (*F*_20,320_ = 35.11, *P* < 0.0001), and whenever differences were significant, family A was more massive than family B.

### Circulating carotenoids

**Figure 4:**
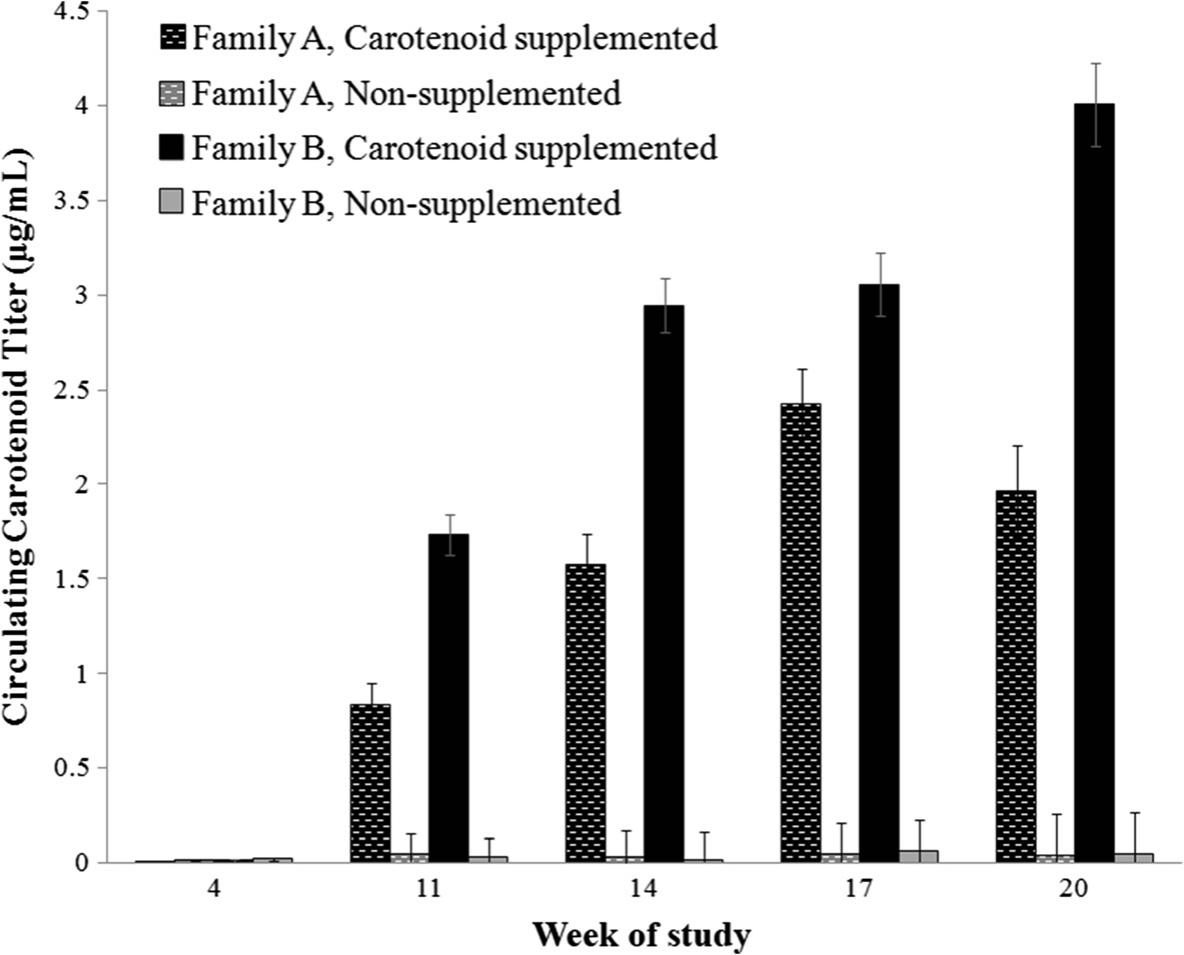
**Circulating carotenoid concentrations measured in growing male veiled chameleons during development.** Before supplementation, there were no significant differences in circulating carotenoid levels among groups. After supplementation began, carotenoid-supplemented individuals had significantly elevated circulating carotenoid concentrations compared to the non-supplemented group. Within carotenoid-supplemented individuals, members of family B always had significantly higher carotenoid titers once supplementation began; however, no such pattern was detected between non-supplemented groups, which circulated barely detectable levels of carotenoids throughout the study. Bar heights represent mean values with standard errors calculated from raw data.

Total circulating carotenoid levels did not differ by family or diet treatment in Week 4, prior to the start of supplementation (Table 1). However, circulating carotenoid levels were significantly different between treatment groups at all subsequent time points (Table 1; Figure 4), such that carotenoid-supplemented individuals circulated higher levels (Week 11, +1.2 μg/mL; Week 14, +2.2 μg/mL; Week 17, +2.7 μg/mL; Week 20, +2.9 μg/mL) of total carotenoids. For comparison, the difference between treatment groups prior to supplementation was 0.008 μg/mL. In addition to these differences, there was an interactive effect of family, treatment, and date on plasma carotenoid concentration, such that family and treatment interacted at multiple time points (Table 1) after supplementation began*. Post hoc* comparisons revealed that families did not differ within the control group (all *P* > 0.898), but family B circulated higher levels of carotenoids than family A in the carotenoid-supplemented group in Weeks 11, 14, and 20 (all *P* < 0.0002; Figure 4). A similar pattern existed in Week 17, though the interaction between family and treatment was not significant.

**Table 1:** Effects of family, treatment, and their interaction on circulating levels of plasma carotenoids (broken down by age)

**Age**	**Independent variable**	***F***	***P***
Week 4	Family	1.24	0.28
	Treatment	1.87	0.19
	Family x Treatment	0.03	0.87
Week 11	**Family**	**16.32**	**0.0011**
	**Treatment**	**131.75**	**< 0.0001**
	**Family x Treatment**	**17.79**	**0.0007**
Week 14	**Family**	**20.36**	**0.0004**
	**Treatment**	**219.41**	**< 0.0001**
	**Family x Treatment**	**21.11**	**0.0004**
Week 17	Family	3.56	0.079
	**Treatment**	**245.36**	**< 0.0001**
	Family x Treatment	3.20	0.094
Week 20	**Family**	**20.73**	**0.0004**
	**Treatment**	**169.45**	**< 0.0001**
	**Family x Treatment**	**20.15**	**0.0004**

Significant terms in bold, and all d.f. = 1,15.

Based on observed morphological differences between families, we hypothesized that body mass was one of the mechanisms driving the family effect on circulating carotenoids in the carotenoid-supplemented group. Therefore, we performed *post hoc* analyses examining the specific effect of mass on plasma carotenoid concentration within treatment groups. While there was no significant effect of mass on carotenoids within treatment groups prior to supplementation (C group *F*_1,8_ = 0.70, *P* = 0.426, estimate = -8.45*10^-4^; N group *F*_1,8_ = 4.70*, P* = 0.062, estimate = -1.30*10^-3^), there was a significant and negative correlation between body mass and carotenoid titer within carotenoid-supplemented individuals at all remaining time points (all *F*_1,8_ > 10.49, all *P* < 0.015, -1.61*10^-2^ < all estimates < -3.66*10^-2^). In contrast, there was no significant correlation between mass and carotenoid titer for non-supplemented individuals at any time point (all *F*_1,8_ > 0.435, all *P* < 0.530, -7.01*10^-5^ < all estimates < -1.42*10^-4^), with one exception. During Week 17, there was a significant relationship in the non-supplemented group between mass and carotenoid titer, with bigger animals circulating a lower concentrations of carotenoids (Week 17; *F*_1,8_ = 5.37, *P* = 0.049, estimate = -6.64*10^-4^). It should be noted that some of these patterns may be due to family effects, as Family A was generally more massive than Family B.

### Agglutination and lysis

**Figure 5:**
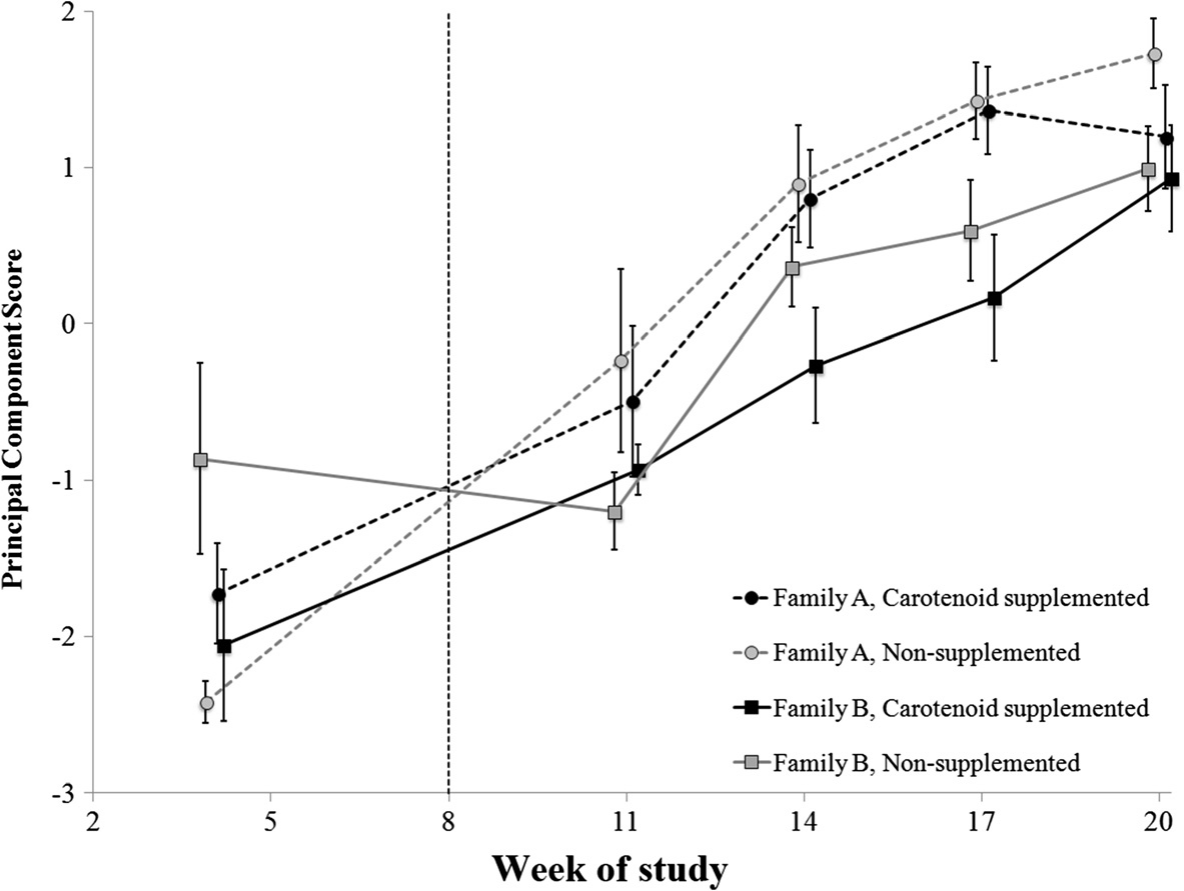
**Agglutination and lysis ability of juvenile veiled chameleon plasma collected during development.** Because agglutination and lysis titers were highly correlated (*r* = 0.98), these two metrics are represented by a unit-less principal component (PC) score, where a higher score indicates (i) enhanced activation of the complement cascade by antibodies, and (ii) greater capacity for complement to lyse foreign cells. Prior to carotenoid supplementation (left of vertical dashed line), there were no significant differences in agglutination and lysis between families or treatment groups. However, family A had significantly higher PC scores than family B at Weeks 14 and 17 of the study. Despite differences based on family of origin, there was no evidence of an effect of carotenoid supplementation on agglutination or lysis. *Post hoc* analyses found no relationship between PC scores and circulating carotenoid levels within treatment group, or between PC score and mass within family. Measurements for all groups were collected at the same times, but data points are offset for ease of interpretation. Points represent mean values with standard errors calculated from raw data.

Overall, there was no significant effect of carotenoid treatment on PC1 for agglutination and lysis (*F*_1,15_ = 0.67, *P* = 0.427); however, there was a significant interactive effect between age and family of origin on this metric (*F*_4,60_ = 3.92, p = 0.007), yielding a transient effect of family on PC1 (Table 2; Figure 5). Specifically, before carotenoid supplementation, families A and B had similar PC1 scores, after which family B had lower PC1 scores than family A until this family effect disappeared in Week 20 (Table 2; Figure 5). Additionally, *post hoc* analyses revealed that there was no relationship within treatment groups between circulating carotenoid levels and PC1 (all *F*_1,7_ < 3.24 , all *P* > 0.121) nor a relationship between mass and PC1 within families (all *F*_1,7_ < 2.64, all *P* > 0.148).

**Table 2:** Effects of family, treatment, and their interaction on the lysis and agglutination response of chameleon plasma (broken down by age)

**Age**	**Independent variable**	***F***	***P***
Week 4	Family	1.55	0.23
	Treatment	0.20	0.66
	**Family x Treatment**	**4.83**	**0.044**
Week 11	**Family**	**4.30**	**0.056**
	Treatment	0.12	0.73
	Family x Treatment	0.09	0.76
Week 14	**Family**	**6.05**	**0.027**
	Treatment	0.79	0.39
	Family x Treatment	0.86	0.37
Week 17	**Family**	**9.81**	**0.0069**
	Treatment	0.58	0.46
	Family x Treatment	0.32	0.58
Week 20	Family	2.18	0.16
	Treatment	1.21	0.29
	Family x Treatment	0.79	0.39

Lysis and agglutination responses were highly correlated and were collapsed into a single principal component score which explained 98% of the variation in these scores. Significant terms in bold, and all d.f. = 1,15.

### Wound healing

**Figure 6:**
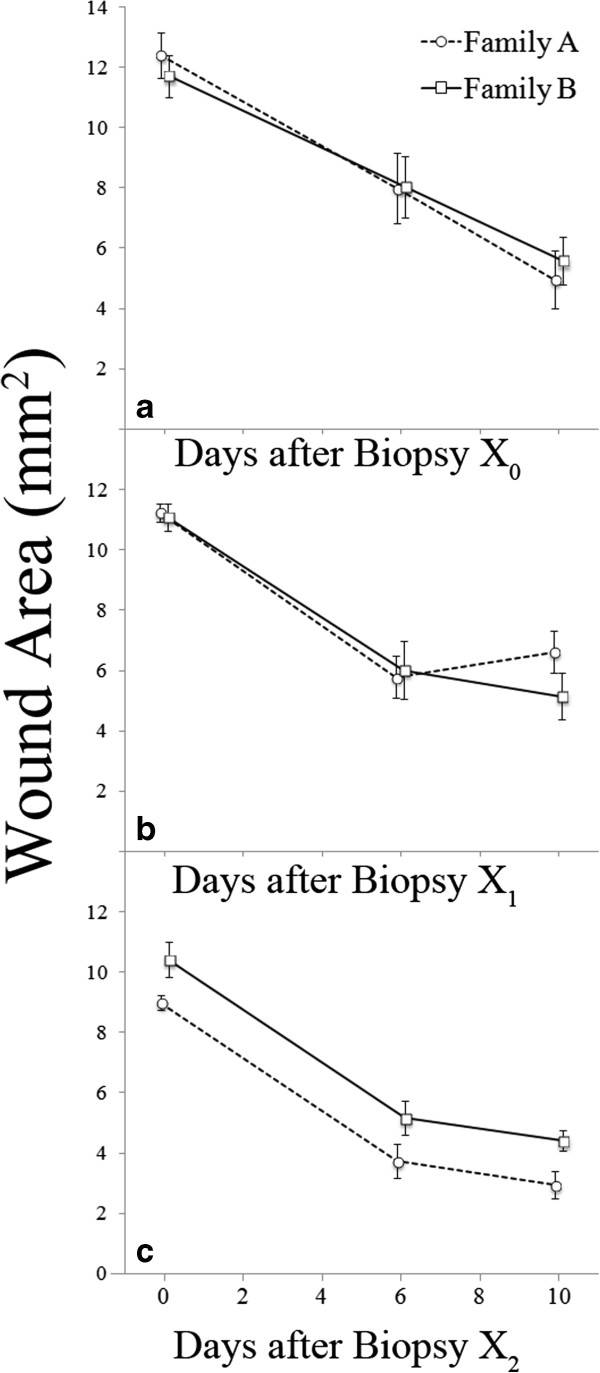
**Wound area of juvenile veiled chameleons immediately (D**_**0**_**), six (D**_**6**_**), and ten (D**_**10**_**) days after a standardized dermal biopsy.** Throughout the course of the study, the chameleons were biopsied three times: once prior to carotenoid supplementation (**a:** Biopsy X_0_) and twice during supplementation in Weeks 14 and 20 of the study (**b:** Biopsy X_1_ and **c:** Biopsy X_2_, respectively). **a)** During X_0_, there was no significant effect of family, treatment, or their interaction (repeated measures: all *F*_1,14_ < 0.97, all *P* > 0.182; mixed model: all *F*_1,30_ < 1.94, all *P* > 0.17). Wound size decreased over time (from D_0_ to D_6_ to D_10_) during X_0_ (repeated measures: *F*_2,28_ = 36.74, *P* < 0.0001; mixed model: *F*_2,30_ = 41.42, *P* < 0.0001). **b)** During X_1_, there was no significant effect of treatment, family, or their interaction on wound healing rate (repeated measures: all *F*_1,12_ < 0.86, all *P* > 0.37; mixed model: all *F*_1,28_ < 1.82, all *P* > 0.19). Again, wound size decreased over time (D_0_, D_6_, D_10_) during X_1_ (repeated measures: *F*_2,24_ = 55.04, *P* < 0.0001; mixed model: *F*_2,28_ = 58.79, *P* < 0.0001). **c)** In contrast to X_0_ and X_1_, during X_2_ there was a significant effect of family of origin on wound size (repeated measures: *F*_1,15_ = 10.60, *P* = 0.005; mixed model: *F*_1,30_ = 10.60, *P* = 0.0028), though again wound size decreased over time (D_0_, D_6_, D_10_; repeated measures: *F*_2,30_ = 96.51, *P* <0.0001; mixed model: *F*_2,30_ = 96.51, *P* <0.0001). Specifically, family A had consistently smaller wounds than family B on all measurement days (D_0_, D_6_, and D_10_) during X_2_. Points represent mean values with standard errors calculated from raw data.

Overall, there were no significant effects of carotenoid treatment, family, or their interaction on wound size (rmANOVA: Family *F*_1,10_ = 0.59, *P* = 0.46; Treatment *F*_1,10_ = 1.57, *P* = 0.24; Family*Treatment *F*_1,10_ *=* 0.97, *P* = 0.35; mixed model: Family *F*_1,119_ = 0.45, *P* = 0.50; Treatment *F*_1,119_ = 2.54, *P* = 0.11; Family*Treatment *F*_1,119_ *=* 0.38, *P* = 0.54; Figure 6). However, whether the wound area measurements were taken after X_0_, X_1_, or X_2_ had a significant influence on wound healing rate (rmANOVA: *F*_2,20_ = 7.85, *P* = 0.003; mixed model: *F*_2,119_ = 22.11, *P* < 0.001). Specifically, for X_0_ all chameleons exhibited an approximately linear trend in wound area reduction, whereas for X_1_ and X_2_ we detected a biphasic healing pattern (Figure 6). Additionally, the initial slope for reduction in wound size was steeper (more negative) for X_1_ and X_2_, indicating faster primary wound healing following these biopsies. (see Figure 6 legend for additional details). Additionally, *post hoc* analyses revealed the following relationships between mass and wound size within family on either Day 6 or Day 10 of wound-healing; Family B showed a trend toward a positive relationship between mass and wound size on Day 10 during both X_1_ (*F*_1,7_ = 5.68, *P* = 0.076, estimate = 0.27) and X_2_ (*F*_1,7_ = 4.72, *P* = 0.066); all other relationships were non-significant (all *F*_1,7_ < 1.54, all *P* > 0.25, -0.16 < all estimates < 0.12).

### Nitric oxide levels

**Figure 7:**
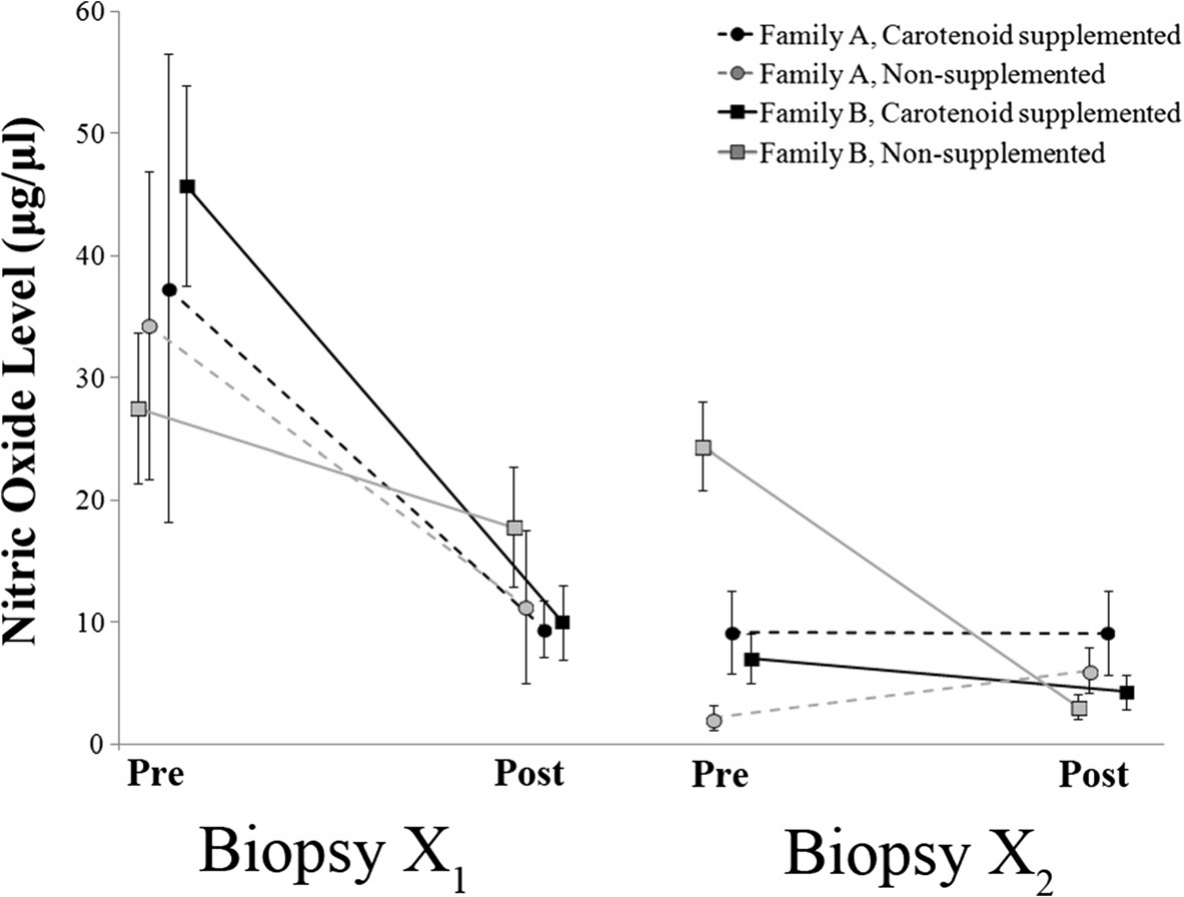
**Circulating nitric oxide (NO) levels in juvenile male veiled chameleons collected immediately before and two days after (data points offset for clarity) dermal biopsies performed during the carotenoid supplementation period (X**_**1 **_**in Week 14 and X**_**2 **_**in Week 20 of the study).** Circulating NO levels decreased from pre- to post-wound, with a much larger drop in X_1_ than in X_2_. In general, there were no detectable treatment effects and NO levels decreased as the chameleons aged. Points represent mean values with standard errors calculated from raw data.

Overall, there were no significant effects of family, treatment, or their interaction on the change in NO levels during both wounding events (Family *F*_1,16_ = 0.64, *P* = 0.435; Treatment *F*_1,16_ = 0.33, *P* = 0.573; Family*Treatment *F*_1,16_ = 0.00, *P* = 0.962). However, there was a significant effect of age at biopsy (Weeks 14 and 20) on change in circulating NO levels (*F*_1,16_ = 7.59, *P* = 0.014). Specifically, NO levels dropped from pre- to post-wounding more for X_1_ than for X_2_ (Figure 7).

There were no significant effects of family, treatment, or their interaction on NO pre- or post-wound in Week 14 (X_1_; Table 3), but there was a significant effect of wounding on NO levels, as NO levels dropped from pre- to post-wound (Table 3). Plasma NO levels from blood collected during Week 20 (X_2_) indicated that there was a significant interaction between treatment, family, and wounding (Table 3). More specifically, in Week 20, three (AC, BC, BN) out of the four groups exhibited decreased NO levels from pre-wounding to post-wounding, and one group (AN) exhibited an increase in NO levels (Figure 7).

**Table 3:** Effects of wounding, family, and carotenoid treatment on circulating nitric oxide (NO) levels (broken down by age)

**Age**	**Independent variable**	***F***	***P***
Week 14	Treatment	0.20	0.66
	Family	0.11	0.74
	Family x Treatment	0.12	0.73
	**Wounding**	**12.58**	**0.0027**
	Wounding x Treatment	1.29	0.27
	Wounding x Family	0.04	0.84
	Wounding x Treatment x Family	0.61	0.45
Week 20	Treatment	0.68	0.42
	Family	2.84	0.11
	**Family x Treatment**	**12.67**	**0.0026**
	**Wounding**	**10.00**	**0.006**
	**Wounding x Treatment**	**5.24**	**0.036**
	**Wounding x Family**	**19.16**	**0.0005**
	**Wounding x Treatment x Family**	**12.46**	**0.0028**

Significant terms in bold, and all d.f. = 1,15.

In *post hoc* analyses, we examined the relationship within treatment groups between circulating carotenoid concentrations and NO levels after wounding within each time point. We found that there were no significant correlations between circulating carotenoid concentrations and NO levels within either the C or N group (all *F*_1,7_ < 0.86, all *P* > 0.383) at either time point (X_1_ or X_2_). This lack of correlation led to a wide range of estimates between circulating carotenoid titer and NO levels (C,X_1_: -2.80; C,X_2_: 385.4; N,X_1_: 1.41; N,X_2_: -28.0).

## Discussion

Individual chameleons supplemented with the carotenoid lutein circulated significantly higher total carotenoid concentrations compared to control animals; however, this treatment did not influence any measured aspect of chameleon immune function. Specifically, there were no effects of lutein supplementation on the agglutination or lysis of SRBCs or on wound healing rate. Additionally, in marked contrast to the general predictions made by Alonso-Alvarez et al. [44] about carotenoids and their ameliorating effects on oxidative stress (such as that due to NO production), we found no effect of lutein supplementation on baseline NO levels or NO burst after wounding. While we uncovered several interesting relationships in this study (e.g., among growth, immunity, and post-wounding NO levels, discussed below), our findings demonstrate that lutein supplementation does not provide any significant immunostimulatory benefit in this species (at least for the immune system attributes we measured).

Selection should favor the evolution of carotenoid-dependent immune function in organisms with regular access to these biomolecules. For example, granivorous, frugivorous, and herbivorous animals that can selectively forage for carotenoid-rich food items (e.g., house finches *Haemorhous mexicanus*[58]; parsnip webworms *Depressaria pastinacella,*[59]; European blackcaps *Sylvia atricapilla*, [60]) seem ideal candidates for the regular use and incorporation of carotenoid molecules into various aspects of the immune response. In contrast, predatory organisms that do not regularly ingest carotenoids directly from plants may be less likely to exhibit strict carotenoid-dependence for essential processes (e.g. immune function; [61]), including ornamental coloration (*cf*[48,62,63]). The lack of any significant effects of lutein supplementation on immune responses of growing veiled chameleons should therefore be cautiously interpreted in a phylogenetic and life-history framework. Specifically, because most chameleons are arthropod generalists [46] and may therefore be limited with respect to how much control they have over the carotenoid content of their food, selection may never have favored evolution of the mechanisms required for carotenoid-based immunoregulation. Carotenoid molecules appear to play an important role in the display colors used by veiled chameleons (authors’ unpublished data); however, our data suggest that incorporation and utilization of carotenoids by veiled chameleons is not required for immune system regulation in this species.

### Family-level influences

During development, family A became significantly more massive than family B in Week 12, a relationship that continued throughout the duration of the study. Unfortunately, this difference resulted in the nesting of body mass within family as well as housing arrangement (all family A cages were positioned higher than family B cages), which confounded our interpretations of all significant family effects that we uncovered. As such, differences between family groups could have arisen due to genetic or early developmental differences (e.g., in growth rate [64]), effects of differing height on microenvironment conditions (e.g., lighting, temperature, humidity [65]), body mass-mediated differences [66], or some interaction among these factors.

One of the primary differences we found between families was that supplemented members of family B always circulated significantly higher carotenoid levels than supplemented members of family A, and this can reasonably be explained by the phenomenon of dose-dilution as a function of body mass [57]. Specifically, a given volume of carotenoids will be more diluted in larger-bodied animals than in those with smaller bodies. That differences between supplemented individuals arose as a result of differences in body mass is further supported by the fact that there were no differences in carotenoid circulation between families before supplementation (at the time when there was no family-based difference in mass) but that such differences existed during three of the four sampling periods after family masses became significantly different (with the fourth sampling period exhibiting the same, albeit non-significant, trend).

To examine performance of the developing humoral arm of the innate immune system, we recorded agglutination and lysis titers at multiple time points during development. We found that agglutination and lysis performance increased as the chameleons aged, but family A’s agglutination and lysis scores were generally higher than family B’s. However, differences in body mass between families did not likely drive the pattern for agglutination, as *post hoc* analyses did not reveal any intrafamilial relationships between agglutination and lysis titers and mass. Given that the proteins that constitute the humoral branch of the innate immune system (i.e., antibodies and the complement system [24,67,68]) are encoded by germ-line genes [68], it is possible that genetic differences drove the observed family difference in these aspects of innate immunity. However, we are unable to test if these family-level differences arose as a result of genetic effects or differences in cage height.

Families A and B had nearly equivalent agglutination and lysis scores by the end of the study (Week 20; Figure 5), a convergence that began in Week 14. Though these chameleons may not have reached full adult size by the end of this study [46], it is possible that, as they progressed into advanced developmental stages, Family A’s immune system was down-regulated, as they approached sexual maturity and began investing in reproductive characters (*cf.* the trade-off model described by [69,70]). Similar trade-offs between reproduction and immune system activity have been documented in great tits *Parus major*[71], crickets *Gryllus texensis*[72], ruffs *Philomachus pugnax*[73], tree lizards *Urosaurus ornatus*[6], and black guillemots *Cepphus grylle*[74]. However, because other variables were correlated with family, a convergence in lysis/agglutination scores due to sexual maturation is just one possibility, and interactions between age and genetic effects or rearing environment may have also influenced the changes in agglutination and lysis scores over time.

Experimental wounding is a biologically relevant immune challenge, and multiple aspects of the immune response can be quantified following the biopsy, making this procedure a valuable tool in investigations of immune system function [30]. Although carotenoid supplementation had no effect on wound-healing in our study, the family of origin had a significant effect on wound size at biopsy X_2_. Specifically, members of family A had consistently smaller wound areas in response to biopsy X_2_ than those of family B, though no differences between families were detected for biopsies X_0_ or X_1_. Oddly, for biopsy X_2_, wound size differed between the two families at all time points, including D_0_. Thus, the intial difference in wound size likely contributed to the observed differences at D_6_ and D_10_ (i.e. healing rate between the two families was similar). Because we used a standardized biopsy procedure with all chameleons, differences in wound area at D_0_ are paradoxical, but might represent a difference in the acute response to trauma (e.g. tissue contractility). Independent of the wound size differences seen in response to biopsy X_2_, it is interesting to note that the pattern of wound healing changed from a linear reduction in wound area over time for biopsy X_0_ (prior to supplementation) to a more biphasic healing trajectory in the subsequent (X_1_ and X_2_) biopsies where healing rate was slower between D_6_ and D_10_ than it was between D_0_ and D_6_. Furthermore, the initial reduction in wound area size was greater (steeper, more negative slope) for biopsies X_1_ and X_2_. Because there were no effects of carotenoid supplementation on wound healing rate, these changes in healing rate may reflect an increased ability to deal with a wound of a given size as the chameleons grow, perhaps because the size of the wound relative to the size of the chameleons was less as the chameleons aged and grew. Additional work is required to determine whether these changes in healing trajectory are linked to age, size, sexual maturation, or some combination of these factors.

### Interpreting long- and short-term changes in NO

Based on previous studies of NO, a key regulator of immune function and a non-specific pathogen destroyer [75], we anticipated that (i) NO levels before wounding would be low and approximately equivalent, representing a baseline circulating NO titer, and (ii) that higher levels would be observed after wounding, reflecting the NO burst triggered by an immune challenge ([33]; reviewed in [34,35]). However, neither of these predictions was met. Instead, we recorded an overall decrease in NO levels in most individuals from pre- to post-wound, regardless of treatment or family group, which is in direct opposition to previous studies in mammals documenting a post-immune challenge increase in NO levels ([33]; reviewed in [34,35]). Unfortunately, because we did not have a non-wounded control group to control for variability in NO related to age or time, our methodological limitations restrict the interpretation of these findings. It is possible that we mistimed our post-biopsy blood collection (though missing peak NO levels does not explain the observed reduction in NO levels from pre- to post-wounding) or that some aspect of the procedure itself caused the unexpected drop in NO. As such, additional work is required to determine the appropriate time course over which to measure NO burst in squamates and the underlying causes of the observed post-wounding decreases in plasma NO levels.

## Conclusions

Dietarily acquired nutrients can play an important role in vital physiological processes, particularly during the energetically demanding period of development and growth. Here, we investigated the possibility that access to supplementary carotenoids during development could influence important immune functions of growing veiled chameleons. However, we found that supplemental carotenoids had no significant influence on the immune system of veiled chameleons at any stage of development. Despite the fact that lutein supplementation did not influence immune function in our experiment, we uncovered several interesting links between life-history and immunity in juvenile male chameleons. An initial increase in immune function (measured in terms of agglutination and lysis ability) throughout the developmental period, followed by a subsequent plateau in this ability, indicates a potentially important interaction between body mass, maturation, and immune function. Additionally, the observed post-wounding decrease in NO levels may represent a difference in the physiological mechanisms and functions of NO in the immune response of chameleons, as compared to that of the endotherms studied to date. Though additional work is required to parse out the relative importance of mass, age, sexual maturity, and microenvironment on the development of immune system function in this species, this study highlights the importance of examining well-studied physiological mechanisms in novel taxa.

## Competing interests

The authors declare they have no competing interests.

## Authors’ contributions

KLM, RAL, and KJM conceived of the study and analyzed carotenoid content from wild-caught food items. RAL and KLM conducted carotenoid supplementation and chameleon husbandry. KLM, KJM, and MWB conducted plasma carotenoid analyses. DFD, KLM, and RAL conducted the wound-healing assays. MWB and KLM conducted the Nitric Oxide analyses and agglutination and lysis assays. All authors helped to draft the manuscript. All authors read and approved the final manuscript.

## Supplementary Material

Additional file 1: Table S1 Wet and dry masses of arthropods collected in July 2011 in LaBelle, FL, USA. Dry masses were obtained after overnight desiccation in a drying oven. The carotenoid content of these arthropods was determined via extraction and HPLC analysis and used to calculate the carotenoid supplementation dose administered to captive veiled chameleons (*Chamaeleo calyptratus*). Click here for file

Additional file 2: Table S2 Correlation coefficients among different circulating carotenoid types. Click here for file

Additional file 3: Figure S1 Photographs illustrating chameleon biopsy (a) and wound-healing at six days (b) and ten days (c) following the biopsy. Original photographs also contained a ruler for scale, which allowed us to calculate wound size after tracing wound outlines (yellow line in b and c) in ImageJ. Note, original wound size was calculated as the outer circumference of the biopsy and was not influenced by slight bleeding noticeable on the right side of the biopsy (a). Click here for file
