# Supplementary material for: The effect of carotenoid supplementation on immune system development in juvenile male veiled chameleons (Chamaeleo calyptratus)
Source: Front Zool. 2014 Mar 22;11:26. doi: 10.1186/1742-9994-11-26 (PMC4022081; doi:10.1186/1742-9994-11-26)
Supplement: Additional file 1: Table S1 — Wet and dry masses of arthropods collected in July 2011 in LaBelle, FL, USA. Dry masses were obtained after overnight desiccation in a drying oven. The carotenoid content of these arthropods was determined via extraction and HPLC analysis and used to calculate the carotenoid supplementation dose administered to captive veiled chameleons (Chamaeleo calyptratus). [file 1742-9994-11-26-S1.doc]

**Additional file 1:** Table S1.

| **Group** | **Number of Individuals** | **Wet Mass (mg)** | **Dry Mass (mg)** | **Total Lutein Concentration^†^** |
| --- | --- | --- | --- | --- |
| *Myriapoda* | 1 | 2147.11 | 560.92 | 10.74 |
| *Blattodia* | 6 | 187.46 | 59.88 | 41.21 |
| *Isopoda* | 4 | 173.00 | 83.22 | 34.14 |
| *Hymenoptera* | 3 | 147.25 | 68.92 | 10.71 |
| *Hemiptera* | 5 | 172.10 | 75.26 | 186.86 |
| *Diptera* | 3 | 97.46 | 31.25 | 62.33 |
| *Orthoptera* | 13 | 1143.01 | 343.32 | 121.75 |
| *Coleoptera* | 6 | 96.30 | 65.03 | 0.79 |
| *Araneae* | 12 | 2202.63 | 755.68 | 2.70 |

**^†^**Micrograms of lutein per gram dry mass
