# Supplementary material for: The effect of carotenoid supplementation on immune system development in juvenile male veiled chameleons (Chamaeleo calyptratus)
Source: Front Zool. 2014 Mar 22;11:26. doi: 10.1186/1742-9994-11-26 (PMC4022081; doi:10.1186/1742-9994-11-26)
Supplement: Additional file 2: Table S2 — Correlation coefficients among different circulating carotenoid types. [file 1742-9994-11-26-S2.doc]

**Additional file 2:** Table S2.

|  | Canary Xanthophyll A | Canary Xanthophyll B | Canary Xanthophyll C | Lutein Isomer | Lutein | Lutein Ester | Zeaxanthin | Total Lutein |
| --- | --- | --- | --- | --- | --- | --- | --- | --- |
| Canary Xanthophyll A |  |  |  |  |  |  |  |  |
| Canary Xanthophyll B | 0.76*** |  |  |  |  |  |  |  |
| Canary Xanthophyll C | 0.88*** | 0.89*** |  |  |  |  |  |  |
| Lutein Isomer | 0.89*** | 0.89*** | 0.98*** |  |  |  |  |  |
| Lutein | 0.88*** | 0.86*** | 0.97*** | 0.97*** |  |  |  |  |
| Lutein Ester | 0.81*** | 0.80*** | 0.92*** | 0.92*** | 0.92*** |  |  |  |
| Zeaxanthin | 0.87*** | 0.91*** | 0.96*** | 0.97*** | 0.98*** | 0.91*** |  |  |
| Total Lutein | 0.88*** | 0.87*** | 0.97*** | 0.97*** | 1.00*** | 0.92*** | 0.98*** |  |
| Total Carotenoids | 0.89*** | 0.88*** | 0.97*** | 0.98*** | 1.00*** | 0.93*** | 0.99*** | 1.00*** |

*** Indicates *p* < 0.001
