# Supplementary figures and images for: The effect of carotenoid supplementation on immune system development in juvenile male veiled chameleons (Chamaeleo calyptratus)
Source: Front Zool. 2014 Mar 22;11:26. doi: 10.1186/1742-9994-11-26 (PMC4022081; doi:10.1186/1742-9994-11-26)

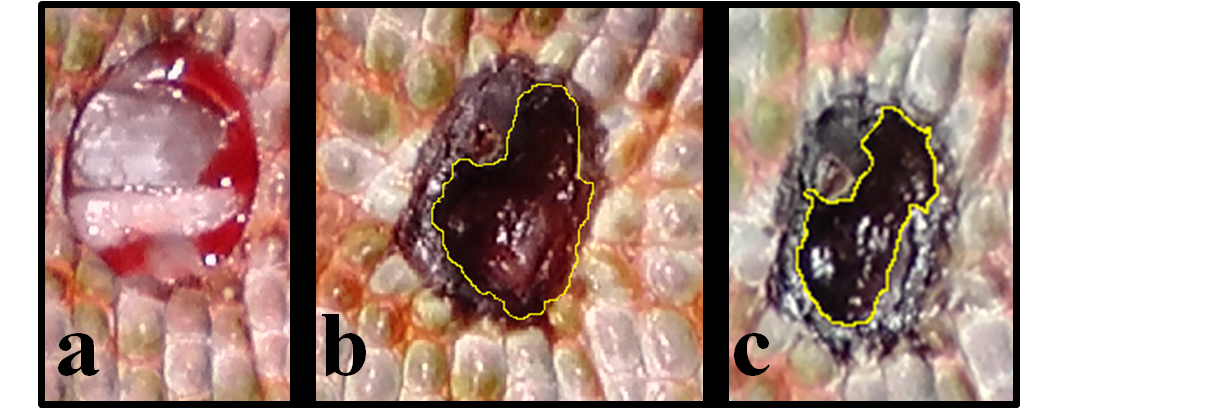
**Additional file 3: Figure S1.**

Supplement: Additional file 3: Figure S1 — Photographs illustrating chameleon biopsy (a) and wound-healing at six days (b) and ten days (c) following the biopsy. Original photographs also contained a ruler for scale, which allowed us to calculate wound size after tracing wound outlines (yellow line in b and c) in ImageJ. Note, original wound size was calculated as the outer circumference of the biopsy and was not influenced by slight bleeding noticeable on the right side of the biopsy (a). [file 1742-9994-11-26-S3.doc]
